# The NRT1.1-NLP7 Nexus: An Integrative Signaling Nexus from Nitrate Sensing to Systemic Adaptation and Structure-Guided Engineering

**DOI:** 10.3390/plants15101539

**Published:** 2026-05-18

**Authors:** Juanxia Chen, Ru Chen, Qian Li, Yihua Zhan

**Affiliations:** 1 National Key Laboratory for Development and Utilization of Forest Food Resources, Zhejiang A&F University, Hangzhou 311300, China; 18293069506@163.com; 2 College of Advanced Agricultural Sciences, Zhejiang A&F University, Hangzhou 311300, China; chenru_0@163.com (R.C.); 19730027686@163.com (Q.L.)

**Keywords:** NRT1.1-NLP7 nexus, nitrate signaling, transceptor, nuclear retention, rational engineering, nitrogen use efficiency

## Abstract

Nitrate functions as both a nutrient and a signaling molecule in plants, initiating genome-wide transcriptional reprogramming and systemic developmental adjustments. Traditionally, plasma membrane nitrate sensing and nuclear transcriptional responses have been considered independent processes linked through linear transduction pathways. However, recent findings reveal that the dual-affinity nitrate transceptor NRT1.1 (NPF6.3) and the transcription factor NLP7 form an integrated signaling nexus—the Nitrate transporter 1.1 (NRT1.1)-NIN-like protein 7 (NLP7) nexus. This review examines the coupling mechanisms, including Ca^2+^-dependent phosphorylation cascades, nucleocytoplasmic shuttling, and a recently discovered MAPK amplification branch. We further explore the nexus’s conserved and diversified functions across crop species, and propose a three-tier rational design framework for reprogramming nitrate responses to enhance nitrogen use efficiency. By bridging structural biology and synthetic biology, this integrative perspective transitions crop improvement from empirical selection to structure-guided design, offering a roadmap for predictive crop engineering.

## 1. Introduction

Nitrogen availability is a primary limiting factor for plant growth and crop productivity. Unlike carbon, which plants acquire from atmospheric CO_2_ via photosynthesis, nitrogen must be sourced from the soil, primarily as nitrate (NO_3_^–^) or ammonium (NH_4_^+^). Nitrate serves not only as a metabolic substrate for amino acid and nucleotide biosynthesis but also as a potent signaling molecule, rapidly influencing genome-wide transcription, developmental programs, and metabolic flux [1,2] within minutes of detection [3,4,5].

Decades of genetic and biochemical studies have elucidated the molecular mechanisms underlying nitrate perception and signal transduction. NRT1.1 (also known as CHL1 or NPF6.3) and NLP7 emerge as key players in the initial perception and transcriptional responses, respectively [4,5,6]. Traditionally, signal transduction was conceived as a linear process: nitrate sensors (NRT1.1) detect ligands, initiate secondary messenger pathways, and activate nuclear effectors (NLP7) [7]. However, recent evidence shows that NRT1.1 and NLP7 form an integrated signaling nexus—the NRT1.1-NLP7 nexus—in which membrane sensing and nuclear transcription are tightly coupled through dynamic phosphorylation networks [8,9]. This nexus operates as a molecular signal integration module, translating variable external nitrate concentrations into specific transcriptional outputs, thereby linking rapid cellular perception with systemic adaptations.

This review focuses on the coupling mechanisms between NRT1.1 and NLP7. We trace signal flow from plasma membrane nitrate sensing, through calcium-dependent phosphorylation cascades, to downstream transcriptional networks regulating root foraging and shoot [9]. By dissecting this core signaling nexus, this review aims to provide a framework for understanding how plants optimize nitrogen acquisition in heterogeneous soil environments and how this knowledge can inform structure-guided engineering strategies.

While previous reviews have separately examined NRT1.1 transceptor biochemistry or NLP7-mediated transcriptional networks [2,10], a mechanistic integration of these spatially segregated processes remains lacking. Specifically, three critical gaps persist in current understanding: (i) how membrane-localized NRT1.1 signaling is temporally decoded to achieve stable nuclear NLP7 activation; (ii) whether the recently discovered direct nitrate sensing by NLP7 [7] operates independently of or synergistically with NRT1.1-mediated calcium signaling; and (iii) how structural insights into conformational switching can inform rational engineering of the entire nexus rather than individual components. This review addresses these gaps by synthesizing structural, biochemical, and systems-level evidence into a unified dual-sensor architecture that positions the NRT1.1-NLP7 nexus as a hierarchical signal integration module. Furthermore, unlike previous agronomic perspectives that treat nitrogen use efficiency as a quantitative trait [11], we propose a three-tier rational design framework grounded in the nexus’s modular architecture—distinguishing evidence-based engineering (Layer 1 variants with validated phenotypes) from exploratory hypotheses (Layer 2–3 designs requiring kinetic validation). This stratified approach provides a roadmap for transitioning from descriptive biology to predictive crop design.

## 2. NRT1.1: Transceptor as Signal Initiator

### 2.1. Structural Basis of Dual-Affinity Transport and Perception

NRT1.1 is a member of the NPF (NRT1/PTR family) proton-coupled oligopeptide transporters (POTs), functioning as a dual-affinity nitrate transporter with high-affinity (HATS, K_m_ ≈ 20–100 μM) and low-affinity (LATS, K_m_ ≈ 5–10 mM) transport modes [4,5]. This affinity switch is regulated by the phosphorylation of Thr101 in the cytoplasmic loop between transmembrane helices 2 and 3. When dephosphorylated, NRT1.1 exhibits high-affinity nitrate binding, while phosphorylation, mediated by the CIPK23-CBL1/CBL9 complex, converts the protein into a low-affinity conformation [4,5].

**Figure 1:**
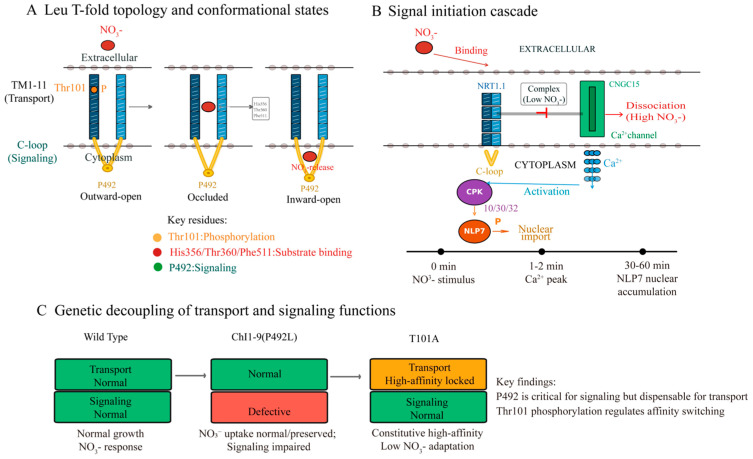
NRT1.1 functions as a dual-function transceptor coupling nitrate transport and signaling. Color code: yellow/orange dots, Thr101 (phosphorylation switch); red dots, substrate-binding pocket residues (His356/Thr360/Phe511); green dots, P492 (signaling loop). (**A**) LeuT-fold topology with critical residues: Thr101 (phosphorylation switch), substrate-binding pocket (His356/Thr360/Phe511), and signaling loop (P492). In panel C, green boxes indicate normal function, red boxes indicate defective signaling, and orange boxes indicate high-affinity locked transport. (**B**) Signal initiation cascade: nitrate binding induces NRT1.1 dissociation from CNGC15, triggering Ca^2+^ influx and CPK activation. (**C**) Genetic decoupling: P492L mutation preserves transport but impairs signaling; T101A mutation locks the high-affinity state.

Crystal structures of bacterial and plant NPF homologues reveal that NRT1.1 adopts a LeuT-fold topology, cycling through outward-open, closed, and inward-open conformations [12]. Substrate binding induces structural rearrangements that couple proton translocation to nitrate transport. Importantly, these conformational changes are not passive; NRT1.1 can detect changes in nitrate concentration and initiate intracellular signaling cascades independent of nitrate transport [4,5], fulfilling the criteria of a transceptor—simultaneously a transporter and receptor with dual functions [2,4] (Figure 1).

### 2.2. Signal Initiation Mechanism

Upon nitrate binding, NRT1.1 induces rapid membrane depolarization and a transient increase in cytosolic calcium (the “nitrate-specific calcium signaling signature”), a critical event for downstream signaling [8,13]. The link between NRT1.1 conformational changes and calcium channel activation was clarified with the identification of the NRT1.1-CNGC15 complex [8]. Nitrate binding triggers NRT1.1 dissociation from CNGC15, activates the CNGC15 channel, and mediates Ca^2+^ influx. Recently, an additional signaling output of NRT1.1 was revealed: under low nitrate conditions, NRT1.1 forms a complex with the receptor-like kinase QSK1 (QSK1 is a negative regulator of PM H^+^-ATPase activity), thereby modulating proton pump activity and extracellular acidification [14]. This NRT1.1-QSK1-H^+^-ATPase module operates in parallel with the CNGC15-calcium branch, expanding the signaling repertoire of the transceptor beyond calcium-centric paradigms.

Additionally, nitrate signaling may interact with other secondary messenger systems, such as phosphatidic acid (PA), diacylglycerol (DAG), and other lipid signals. However, the direct link between these lipid messengers and NRT1.1 remains to be fully explored. These lipids could recruit protein kinase C-like activities (CPKs in plants) to the membrane, initiating cytosolic phosphorylation cascades [9]. The nuclear targets of these cascades are detailed in Section 4.1.

### 2.3. Decoupling of Transport and Signaling

Genetic profiling of alleles defective in transport but retaining signaling function (or vice versa) has demonstrated that NRT1.1’s transport and signaling functions are genetically separable [4,5]. Mutations affecting the proton-coupling mechanism (e.g., in the conserved ExxER motif) abolish transport without affecting signaling, whereas mutations in the C-terminal intracellular loop (e.g., P492L, chl1-9 (P492L mutation in the C-terminal intracellular loop)) impair signaling while preserving transport activity [4]. This functional modularity suggests that NRT1.1 operates in distinct but overlapping conformational states for substrate transport and signal initiation. This concept is essential for understanding how plants decouple sensing from consumption during nitrogen starvation—enabling them to detect nitrate and trigger adaptive responses without excessive nitrate uptake. It also provides a foundation for independently optimizing transport and signaling functions in structure-guided engineering.

## 3. NLP7: A Nuclear Gateway for Nitrate Responses

### 3.1. Molecular Architecture and DNA Recognition

NLP7 is a member of the RWP-RK family of transcription factors, characterized by the conserved RWPxRK motif (Arg-Trp-Pro-x-Arg-Lys), which is essential for DNA binding [7]. The protein consists of an N-terminal inhibitory domain, a central RWP-RK DNA-binding domain that recognizes nitrate response elements (NRE: (A/T)NN(A/C)GNN(A/C)TN), and a C-terminal PB1 (Phox and Bem1) domain responsible for mediating protein–protein interactions [6,7].

Structural modeling suggests that NLP7 binds DNA as a dimer, with the RWP-RK domain making specific contacts with the major groove of NRE-containing promoters. The PB1 domain facilitates homo- and heterodimerization with other NLP family members (NLP6, NLP8) and likely mediates interactions with co-activators of the basal transcription machinery [9,10].

### 3.2. Nucleocytoplasmic Shuttling Dynamics

In the absence of nitrate, NLP7 shuttles continuously between the nucleus and cytoplasm, with a predominant cytoplasmic localization due to the nuclear export machinery, specifically CRM1/Exportin-1, which recognizes the leucine-rich nuclear export signal (NES). Upon nitrate stimulation, NLP7 undergoes rapid nuclear retention, a critical activation step for transcriptional function [6].

Nuclear retention is regulated by the phosphorylation of serine residues, such as Ser205 in the N-terminal region, by CPK10/30/32 [9]. Phosphorylation either masks the NES or enhances the nuclear localization signal (NLS), thereby favoring nuclear accumulation. Live-cell imaging using NLP7-GFP fusion proteins revealed that nuclear retention occurs within minutes after nitrate resupply, peaking between 30 min and 1 h [6], prior to maximal transcriptional activation.

### 3.3. “Hit-and-Run” Transcription Dynamics

NLP7 adopts a dynamic binding mode at target gene promoters, rapidly cycling between binding and dissociation, with the cumulative binding frequency determining the transcriptional output [6,9]. Rather than forming a stable transcription complex, NLP7 undergoes rapid cycles of association and dissociation with DNA, ensuring rapid responses to fluctuating nitrate concentrations and preventing promoter saturation. The PB1 domain facilitates transient interactions with transcriptional co-activators [6,10], while specific phosphatases, whose identities require further investigation, reset the system by dephosphorylating NLP7 [6,9].

## 4. Nexus: Coupling Mechanism Between NRT1.1 and NLP7

The functional coupling between NRT1.1 and NLP7 forms a central integrative signaling nexus that converts extracellular nitrate signals into nuclear transcriptional responses. This coupling is mediated by several interrelated mechanisms:

### 4.1. Calcium-CPK Relay

The most well-characterized coupling mechanism involves Ca^2+^-dependent signal relay initiated by membrane-localized NRT1.1. However, as detailed in Section 4.5, this represents only one component of a hierarchical dual-sensor system that incorporates direct intracellular sensing by NLP7. Activation of NRT1.1 induces cytosolic calcium transients that, in turn, activate CPKs (CPK10, CPK30, CPK32), which directly phosphorylate NLP7 at multiple serine residues. This phosphorylation is necessary and sufficient for nuclear retention [6]. The temporal hierarchy—Ca^2+^ peaks within minutes [13], phosphorylation at 10–20 min [9], nuclear accumulation is maximal at 30–60 min [6]—establishes a ‘calcium decoding’ mechanism.

The temporal dynamics of this process are critical: Ca^2+^ peaks occur within minutes [13], followed by peaks in NLP7 phosphorylation [9], with nuclear accumulation reaching its maximum between 30 min and 1 h [6,9]. Transcriptional activation peaks after 1–2 h. This hierarchical timeline suggests a “calcium decoding” mechanism, where CPKs translate dynamic calcium signals into stable phosphorylation states, enabling precise control of transcriptional output [9].

### 4.2. Direct Phosphorylation Network

While CPKs are the primary mediators of this coupling, other kinases may fine-tune the nexus. For example, phosphorylation of NRT1.1 Thr101 by CIPK23 not only regulates its transport affinity but may also modulate its signaling function, indirectly influencing the output of the NRT1.1-NLP7 nexus [4,5]. Additionally, specific phosphatases likely dephosphorylate NRT1.1 and NLP7, indicating a coordinated mechanism for signal termination [9].

#### 4.2.1. MAPK Cascade: A Parallel Phosphorylation Branch for Nitrate Signal Amplification

A newly discovered MAPK cascade operates in parallel with the CPK-NLP7nexus, providing an additional phosphorylation layer for nitrate signal amplification. Recent studies have demonstrated that nitrate resupply triggers, within minutes, the activation of an MKK3-dependent MAPK module composed of MAP3K13/14, MKK3, and clade-C MAPKs (MPK1/2/7/14) [14]. Critically, this cascade is initiated by NLP-dependent transcriptional induction of MAP3K13 and MAP3K14—NLP7 directly binds to their promoters—establishing a feedforward loop where NLP7 not only activates immediate transcriptional responses but also induces kinase cascades that modulate nitrate uptake and senescence. The nitrate-induced MPK7 activation occurs independently of nitrate reduction (nitrate reductase-deficient mutants show normal activation), confirming that nitrate itself, rather than downstream metabolites, serves as the signal. Notably, MPK7 does not phosphorylate NLP7 directly, suggesting that this MAPK branch modulates nexus output through distinct substrates rather than direct NLP7 modification, expanding the phosphorylation network beyond the established CPK10/30/32-Ser205 nexus.

#### 4.2.2. MKK3-Dependent MAPK Module: Context-Specific Functions Across Developmental Stages

The MKK3-dependent MAPK module represents a second phosphorylation branch that is activated by nitrate and elicits distinct physiological outputs. In seeds, MAP3K13/14/19/20-MKK3-MPK1/2/7/14 is activated by nitrate and light to modulate secondary dormancy release, operating independently of the canonical ABA/GA hormone balance [15]. In roots, the same module forms a positive feedback loop with the circadian clock component CCA1, enhancing auxin signaling and sustained lateral root elongation into nitrate patches [16]. This functional versatility—dormancy release in seeds, nitrate uptake kinetics in seedlings, and foraging plasticity in roots—highlights the evolutionary repurposing of MAPK modules across developmental contexts, with nitrate serving as a common activating signal.

### 4.3. Metabolic Feedback and “Delayed” Signaling

Beyond rapid phosphorylation events, metabolic intermediates provide slower feedback regulation. Nitrate assimilation generates glutamine and asparagine, which exert negative feedback inhibition on NRT1.1 expression [3]. These metabolites may also influence the expression of nitrogen-responsive genes via metabolite sensing. The TOR (target of rapamycin) kinase complex, which integrates signals related to cellular energy and carbon status, may coordinate with the NRT1.1-NLP7 nexus through parallel pathways or downstream effectors [17,18]. This coordination ensures that anabolic programs are activated only when sufficient carbon skeletons are available to support amino acid synthesis.

A newly discovered epigenetic layer adds temporal precision to this metabolic feedback. Bellegarde et al. (2025) demonstrated that fluctuating nitrate availability dynamically reshapes the chromatin landscape of IPT3—the key cytokinin biosynthesis gene—through antagonistic histone H3 modifications [19]. Under nitrate starvation, the repressive mark H3K27me3 is deposited by CLF-containing PRC2 complexes while the activating mark H3K4me3 is removed by JMJ14 demethylase, locking IPT3 in a transcriptionally silent state. Upon nitrate resupply, H3K27me3 demethylation mediated by REF6/ELF6/JMJ13 unlocks IPT3 within minutes, followed by NLP7-dependent H3K4me3 deposition via ATX1 that sustains transcriptional activation. This chromatin-mediated “molecular memory” enables plants to distinguish transient nitrogen fluctuations from sustained nutrient availability, preventing premature cytokinin-driven shoot growth when nitrate supply is unstable.

### 4.4. Carbon-Nitrogen Coupling and Metabolic Gating

Although NRT1.1 is primarily localized to the plasma membrane, the close proximity of the endoplasmic reticulum (ER) to the nucleus may facilitate rapid signal transduction to the nucleus via multiple pathways.

The cytosolic calcium signal induced by NRT1.1 is not a simple binary switch but rather a dynamic pattern with intricate temporal characteristics [13]. Calcium signals with varying frequencies and amplitudes can potentially lead to distinct activation patterns of CPKs, which, in turn, influence the phosphorylation dynamics and transcriptional output of NLP7 [9]. This frequency-dependent response, if confirmed, would enable the system to differentiate between transient nitrogen fluctuations and sustained changes in nitrogen availability. Mathematical modeling suggests that the interaction between CPKs and phosphatases in a positive feedback loop could yield nonlinear response behaviors, although this hypothesis requires experimental validation.

The nexus output is not only determined by nitrate input but is also tightly regulated by intracellular carbon metabolism. Recent studies indicate that SnRK1 kinase directly phosphorylates Ser125 and Ser306 of NLP7 during carbon deficiency, promoting its cytoplasmic retention and degradation, thereby inhibiting anabolic processes [20]. The TOR kinase, which monitors cellular energy and carbon status, governs global anabolic pathways [17,18]; however, its direct relationship with NLP7 remains to be established. While SnRK1 directly phosphorylates NLP7 at Ser125/306 to promote cytoplasmic retention [20], the TOR-NLP7 interaction remains elusive, though TOR likely antagonizes SnRK1 to modulate NLP7 activity through indirect phosphorylation cascades. This carbon-nitrogen coupling maintains anabolic energy efficiency.

Additionally, a newly identified mechanism positions NLP7 as a direct regulator of plastid carbon metabolism under nitrogen limitation. That low nitrogen stress induces NLP7 binding to the promoter of plastid glucose-6-phosphate dehydrogenase 3 (G6PD3), a key enzyme in the oxidative pentose phosphate pathway (OPPP) that generates NADPH and ribose for anabolic processes [21]. NLP7-dependent G6PD3 activation serves dual functions: (i) maintaining NADPH/ROS redox homeostasis to protect root meristem integrity, and (ii) suppressing cytokinin biosynthesis (via downregulation of CYP735A1 and upregulation of CKX1/5/6) to inhibit excessive cell division under energy-limited conditions. This NLP7-G6PD3-cytokinin axis operates in parallel with the SnRK1-TOR module, providing an additional layer of carbon-nitrogen metabolic integration that prioritizes root growth maintenance over shoot biomass accumulation during nitrogen starvation [21].

### 4.5. Dual-Sensor Architecture: Integrating Membrane and Nuclear Nitrate Perception

Recent advances have expanded the conceptual framework of nitrate perception beyond membrane-localized sensing by NRT1.1. That NLP7 functions intrinsically as a cytosolic nitrate sensor, binding nitrate through a conserved allosteric pocket distinct from its DNA-binding RWP-RK domain [7]. This challenges the conventional linear model wherein NRT1.1 serves as the sole sensor, raising a critical question: how do spatially segregated sensors coordinate to generate coherent transcriptional outputs?

We posit that the NRT1.1–NLP7 nexus operates as a hierarchical dual-sensor system with specialized temporal and spatial roles. At the plasma membrane, NRT1.1 functions as a rapid initiator, generating calcium signatures within minutes through CNGC15 activation [8] (Section 2.2). Concurrently, cytosolic nitrate is directly sensed by nuclear-localized NLP7, serving as a persistence detector ensuring sustained activation. Nitrate binding to NLP7 unmasks its nuclear localization signal, promoting nuclear retention [6]. This architecture confers three advantages: (i) speed—rapid calcium signaling; (ii) fidelity—nuclear sensing filters transient fluctuations; and (iii) amplification—convergent inputs onto CPK10/30/32 generate cooperative kinetics [9]. This reconciles NRT1.1-independent responses in chl1 mutants, now attributed to direct NLP7 sensing of cytosolic nitrate via alternative transporters (e.g., NRT2.1) [7].

This dual-sensor system explains the biphasic Primary Nitrate Response: an initial NRT1.1-dependent wave (20–30 min) followed by an NLP7-sustained phase (1–2 h) [1,6], reflecting evolutionary optimization for rapid adaptation and stable metabolic reprogramming. Validation requires structural elucidation of the NRT1.1-CNGC15 heterocomplex at the plasma membrane and nuclear NLP7 in complex with CPK10/30/32 and DNA, ideally through cryo-electron microscopy and AlphaFold2-Multimer modeling, respectively.

## 5. Downstream Networks and Physiological Outputs

The NRT1.1-NLP7 nexus coordinates a hierarchical transcriptional cascade that governs physiological adaptation across multiple scales:

### 5.1. Primary Nitrate Response (PNR)

The Primary Nitrate Response (PNR) exhibits characteristic biphasic transcriptional dynamics (Section 4.5): an initial wave peaking at 20–30 min driven by NRT1.1-mediated calcium signaling, followed by an NLP7-sustained phase maximal at 1–2 h. Within this framework, NLP7 activates hundreds of genes involved in nitrate assimilation (e.g., NIA1, NIR), high-affinity transport (e.g., NRT2.1, NRT2.2), and metabolism [6]. These “immediate-early genes” constitute the first tier of the transcriptional cascade. NLP7 also activates secondary transcription factors, such as SPL9 and TGA1/4, which propagate signals to downstream genes that regulate cell division and hormone signaling [22,23]. Additionally, TCP20 and NAC family members act as parallel or downstream regulatory factors, further contributing to the hierarchical transcriptional regulation of the nitrate response.

### 5.2. Plasticity of Root System Structure

Localized high-nitrate patches stimulate lateral root elongation via an NRT1.1-dependent pathway [4,24]. This process is primarily mediated by the transcription factor ANR1, which promotes lateral root growth into nitrate-rich soil regions [1]. At the same time, systemic signaling adjusts to the nitrogen status of the entire plant: when nitrogen levels are sufficient, cytokinin, synthesized in the shoots via IPT3/5/7, is transported to the roots through the xylem. This inhibits lateral root initiation, optimizing foraging efficiency [25,26].

### 5.3. Aboveground Growth and Carbon Allocation

Through systemic signaling, NLP7-mediated nitrate signaling enhances cytokinin biosynthesis in roots via upregulation of IPT3 [27]. This cytokinin is then transported to the shoot, stimulating cell division and leaf expansion. Moreover, NLP7 directly regulates genes related to the photosynthetic apparatus (e.g., PEPC, ICDH, VAR2), ensuring that carbon fixation aligns with nitrogen assimilation capacity [6,28]. This coordination prevents nitrogen accumulation without sufficient carbon skeletons, maintaining an optimal carbon-to-nitrogen ratio [1].

### 5.4. Feedback Conditioning and Signal Termination

To prevent overstimulation, the nexus incorporates multiple negative feedback loops. Prolonged high nitrogen supply inhibits the expression of high-affinity transporter genes, such as NRT2.1, via NRT1.1-dependent signaling (phosphorylated form), establishing a negative feedback loop [3]. Natural variation in NRT1.1B, the rice ortholog of NRT1.1, contributes to nitrate-use divergence between rice subspecies, highlighting the evolutionary conservation and agricultural relevance of this regulatory mechanism [29]. Additionally, the CEP (C-terminal encoded peptide) family, expressed under nitrogen limitation, is transported to the shoot via the xylem. Upon binding to the CEPR1/2 receptor (CRA2 in legumes), it triggers the production of CEPD polypeptides, which return to the roots through the phloem and upregulate nitrate transporter genes like NRT2.1, forming a long-distance feedback mechanism that integrates the nitrogen status across the entire plant [1].

## 6. Nexus in Crops: Functional Conservation Versus Diversification

### 6.1. Rice: Dual NRT1.1 System

Rice contains two NRT1.1 homologous genes, OsNRT1.1A and OsNRT1.1B, which originated from Poaceae-specific genome duplications [30]. OsNRT1.1A is constitutively expressed in root tips, whereas OsNRT1.1B is inducible, showing allelic variation between indica and japonica subspecies [29,30]. The indica allele of OsNRT1.1B exhibits higher nitrate transport activity and signaling efficiency, contributing to better nitrogen use efficiency (NUE) in indica varieties compared to japonica [29]. Overexpression of both OsNRT1.1A and OsNRT1.1B significantly enhances yield and NUE under both low and high nitrogen conditions [30].

The NLP homologs in rice, OsNLP3 (the closest relative to Arabidopsis NLP7) and OsNLP4, play pivotal roles in determining tiller number and grain yield [31]. OsNLP4 transcriptionally represses the strigolactone receptor component OsD3 by binding to the NRE element in its promoter, thereby attenuating strigolactone signaling and promoting tillering [31]. Both OsNLP3 and OsNLP4 regulate panicle development and the number of grains per panicle by activating the transcription factor OsRFL, which increases yield [31]. Additionally, DEP1 (Dense and Erect Panicle 1), a G protein signaling component, regulates plant height, panicle density, and nitrogen response through independent pathways [11]. It works in concert with OsNLP3/4 to influence nitrogen use efficiency [11,31].

### 6.2. Maize and Wheat: Polyploid Complexity

In maize, the genome contains four NRT1.1 homologous genes (ZmNRT1.1B/C/D, etc.), with ZmNPF6.6 (ZmNRT1.1B) identified as a pH-dependent high-affinity nitrate transporter [32]. ZmNPF6.4, on the other hand, exhibits different substrate specificity and chloride ion sensitivity [32]. However, the specific functional roles of these genes in nitrate sensing and signal transduction, their tissue-specific expression patterns, and interactions with the NLP family remain to be thoroughly explored.

In allohexaploid wheat, the NPF family has undergone significant expansion, with more than 200 members [33,34]. NRT1.1-family genes exist as homeologous triads across the A, B, and D subgenomes, with each triad derived from the corresponding chromosomes of the three ancestral genomes, exhibiting differential expression patterns, such as selective polyadenylation [33,34]. However, the functional differentiation, retention, and loss patterns of the NRT1.1-NLP nexus across different subgenomes, as well as its contribution to nitrogen use efficiency, require further investigation. The subfunctionalization of homeologous NRT1.1 copies in wheat remains elusive, particularly regarding dosage compensation effects and dominance expression bias among the A/B/D subgenomes, which are critical determinants of nitrogen use efficiency in polyploid crops.

### 6.3. Breeding Applications

Natural variation in the promoter regions of NRT1.1 and NLP7 underpins differences in nitrogen use efficiency among germplasm resources [29]. Editing the Thr101 site of NRT1.1 allows for regulation of its affinity: the T101A (phosphorylation-defective) locks the transporter in a low-affinity mode, reducing luxury nitrogen uptake in high-nitrogen environments; the T101D phosphomimetic mutation locks the transporter in a high-affinity mode [4,5], improving nitrogen uptake under low-nitrogen conditions. Similarly, constitutive activation of nitrogen assimilation genes can be achieved by editing the NES of NLP7 (e.g., at the L299 site), enhancing nuclear retention [6]. However, such modifications require strict spatial control, such as a root-specific promoter, to prevent ectopic expression that could disrupt the plant’s overall metabolic balance.

### 6.4. Evolutionary Trajectory and Ecological Adaptation of Axial Variation

Through comparative genomic analysis, the core components of the NRT1.1-NLP7 nexus were traced back to the origin of land plants [35]. It is hypothesized that fast-growing plants, such as *Arabidopsis*, may exhibit lower thresholds for axial response to better adapt to fluctuating nitrogen supplies, while perennial crops, like apples, may have higher thresholds to prevent excessive nitrogen uptake. However, these hypotheses require experimental validation.

The nexus’s capacity for signal integration extends beyond nitrogen sensing to coordinate with other environmental cues. Regnard et al. (2024) reported that the MKK3-dependent MAPK module—composed of MAP3K13/14/19/20, MKK3, and clade-C MAPKs (MPK1/2/7/14)—functions as a signal integrator that converges nitrate and light inputs to modulate seed secondary dormancy [15]. The conservation of the MKK3-MAPK module across diverse physiological contexts—seed dormancy [15], nitrate uptake kinetics [14], and root foraging plasticity [16]—underscores its evolutionary importance as a conserved phosphorylation layer within the broader nitrate signaling network. Natural variation in MEKK14 promoter strength among *Arabidopsis* accessions correlates with differential root foraging capacity [16], suggesting that kinase cascade components represent untapped targets for crop adaptation to variable nitrogen environments [23].

## 7. Structural and Synthetic Perspectives: Structure-Guided Engineering and Field Deployment of the NRT1.1-NLP7 Nexus

**Figure 2:**
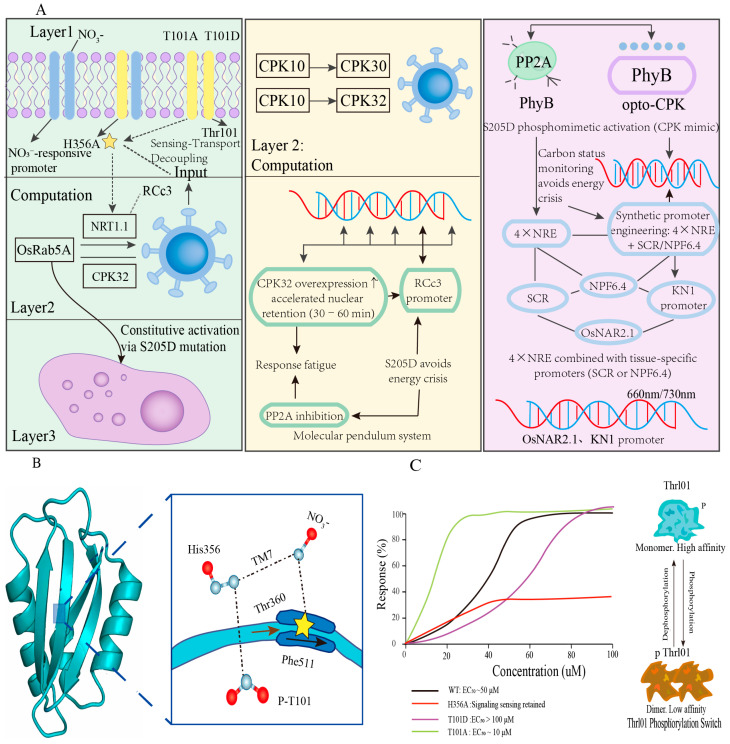
Hierarchical structure-guided engineering framework of the NRT1.1-NLP7 nexus. Evidence stratification: Green-shaded variants are experimentally validated; yellow-shaded strategies are model-based hypotheses; purple-shaded designs integrate validated mechanisms with untested concepts; gray borders indicate theoretical proposals requiring validation. (**A**) Three-tier rational engineering strategy. Input layer (green): NRT1.1 membrane localization with sensitivity-tuning variants (T101A high-affinity hypersensitive [9,12,38], H356A signal-transducing [5,12]); Computational layer (yellow): CPK-PP2A molecular pendulum with dynamics reprogramming (CPK32 overexpression [9], PP2A inhibition [39], opto-Nexus [40,41]); Output layer (purple): NLP7 nuclear localization with transcriptional optimization (S205D constitutive active, synthetic promoters [42,43]). Arrow thickness indicates signal intensity; asterisks mark rationally designed mutations. (**B**) AlphaFold2-Multimer model of NRT1.1 (**left**) and substrate-binding domain (**right**). His356 (TM7), Thr360, and Phe511 form a hydrogen-bonding network for NO_3_^−^ recognition (red) [12]; Thr101 phosphorylation (P-T101, yellow asterisk) regulates conformational switching [9,12,38]. (**C**) Predicted dose–response curves of rationally designed mutants. Dose–response relationships were modeled by the Hill function with cooperative binding (nH = 1.5–2.0), and EC_50_ values were derived from published electrophysiology and yeast complementation data [12,36]. Wild-type (WT, black) exhibits a sigmoidal curve with EC_50_ ~50 μM, operating between HATS and LATS modes [12,36,38]. T101A (green) shows a left-shifted threshold (EC_50_ ~10 μM), locking NRT1.1 in the dephosphorylated high-affinity monomeric state [12,38]. T101D (purple) shows a right-shifted threshold (EC_50_ > 100 μM), mimicking the CIPK23-phosphorylated low-affinity dimeric state [4,12]. H356A (red) loses high-affinity transport while retaining signal-sensing function (undefined EC_50_, transport-abolished/signaling-retained, validated by P492L phenocopy [5]). Inset: Thr101 phosphorylation switch. Dephosphorylation drives the monomeric high-affinity state; phosphorylation drives the dimeric low-affinity state [12,36].

The modular architecture of the NRT1.1-NLP7 nexus presents a systematic entry point for structure-guided engineering. Leveraging the structural model predicted by AlphaFold2-Multimer [36,37] and the conserved folding characteristics of the NPF family [12,32], a three-level engineering strategy is proposed, targeting input sensing, signal computation, and transcriptional output (Figure 2) [5,6,12].

### 7.1. Structure-Based Hierarchical Engineering Strategy

Evidence stratification framework: The engineering strategies presented below are explicitly stratified by evidence maturity to distinguish actionable designs from exploratory hypotheses:

Layer 1 (Input sensing): [Validated]—Grounded in experimentally characterized mutant phenotypes with published transport/signaling assays [4,5].

Layer 2 (Signal computation): [Model-Based Hypothesis]—Extrapolates from established phosphorylation dynamics [4,9] but requires direct kinetic validation; no published mutants currently exist.

Layer 3 (Transcriptional output): [Partially Validated]—Integrates proven nuclear retention mechanisms with untested spatiotemporal control elements [6].

Cross-layer epistasis: [Predictive Modeling]—Derived from published mutant phenotypes and pathway analysis [4,5,6] but represents predictive extrapolations.

This tiered approach guides prioritization for experimental validation and resource allocation.

Based on the modular architecture of the NRT1.1-NLP7 nexus, we propose a hierarchical engineering framework illustrated in Figure 2, targeting three functional layers: input sensing (Layer 1), signal computation (Layer 2), and transcriptional output (Layer 3). This design leverages the structural bistability of NRT1.1 (Section 2.1) [4,5], nucleocytoplasmic shuttling dynamics of NLP7 (Section 3.2) [6], and the CPK-mediated phosphorylation cascade (Section 4.1) [9].

#### 7.1.1. Layer 1: Input Layer Transformation—Rational Tuning of Nitrate Sensitivity Threshold

##### Evidence Status: [Validated]

The following variants are grounded in experimentally characterized mutant phenotypes. Transport kinetics and signaling outputs have been quantified in heterologous systems or mutant lines [5].

The substrate recognition of NRT1.1 is mediated by a hydrogen bond network formed by His356 (TM7), Thr360, and Phe511 [12], along with conformational changes regulated by the phosphorylation state of Thr101 [4,5]. Structure-guided rational design can enable precise regulation of affinity through the following strategies:

##### High-Threshold Nexus

P492L (chl1-9) mutation: [Validated Phenotype]. This mutation disrupts transport activity but preserves the signal-sensing function [5]. This achieves “sensing-transport” decoupling, making it suitable for studying pure signaling functions [5,16]. Note: The P492L mutant was originally isolated in a genetic screen for nitrate signaling defects; transport abolition was confirmed by electrophysiology [5,6].

T101A (phosphorylation-defective): [Validated Phenotype] This mutation locks NRT1.1 in the low-affinity dimer state, reducing excessive nitrate uptake in high-nitrogen environments and promoting the transition to different growth stages. Note: T101A was generated by site-directed mutagenesis and characterized in Xenopus oocytes; it phenocopies the chl1-5 allele, showing abolished HATS activity [12,16,44].

##### Low-Threshold Nexus

T101D mutation (phosphorylation mimic): [Validated Phenotype] By introducing negative charges, this mutation simulates the phosphorylation state induced by CIPK23, disrupting the dimer interface and locking NRT1.1 in the high-affinity monomer conformation [12,16]. This enhances foraging capacity and root plasticity in low-nitrogen environments [12,13]. Note: T101D approximates the CIPK23-CBL1/9-activated phosphorylated monomeric state; however, direct electrophysiological validation in planta remains to be performed [10,12].

The predicted EC_50_ values were benchmarked against experimentally validated states: T101D (10 μM) approximates the phosphorylated monomer induced by CIPK23 overexpression [13], while T101A (>500 μM) phenocopies the chl1-5 transport-defective mutant [12,16]. Direct electrophysiological validation in Xenopus oocytes or yeast complementation assays remains to be performed for synthetic variants combining multiple mutations.

#### 7.1.2. Layer 2: Computational Layer Transformation—Rational Reprogramming Signal Dynamics

##### Evidence Status: [Model-Based Hypothesis]

The following proposals extrapolate from established phosphorylation dynamics but require direct kinetic validation. No published mutants or transgenic lines currently exist for these specific modifications [4,6,9].

Conceptual design: The information processing capacity of the NRT1.1-NLP7 nexus is determined by the dynamic balance between CPK-mediated phosphorylation and phosphatase-mediated dephosphorylation [6,9]. Optimizing this “molecular pendulum” system can enhance the nexus’s response characteristics:

##### Accelerated Response Type

CPK32 overexpression: [Hypothesis; Testable]. Overexpression of CPK32 enhances NLP7 Ser205 phosphorylation [9], potentially accelerating nuclear retention (from 30 to 60 min to <15 min) [6]. This is ideal for environments with rapid nitrogen fluctuations, but may sacrifice adaptive memory, resulting in response fatigue. Established fact: CPK32 phosphorylates NLP7 at Ser205 in vitro and in vivo [9]. Hypothetical extension: The kinetic consequences of CPK32 overexpression on nuclear retention timing have not been experimentally quantified; predictions are based on first-order enzyme kinetics extrapolation.

##### Continuous Activation Type

Phosphatase inhibition: [Hypothesis; Speculative] Inhibition of protein phosphatases that dephosphorylate NLP7 could theoretically prolong nuclear retention and enhance transcriptional output [39]. This approach must be confined to root cap tissue (e.g., using the RCc3 promoter [45]) to prevent metabolic imbalance due to ectopic expression [45,46]. Established fact: NLP7 dephosphorylation resets the signaling system [6,9]. Hypothetical extension: The specific phosphatases (PP2A, PP2C [39,46], or other Ser/Thr phosphatases) responsible for NLP7 dephosphorylation remain unidentified; candidate predictions are based on bioinformatic analysis of serine/threonine phosphatase families [39] and analogy to animal calcium signaling [47].

##### Optogenetic Control Type

Opto-CPK photocontrol: [Theoretical; Proof-Of-Concept Needed] Adapting optogenetic strategies originally developed for animal cells (e.g., PhyB-PIF6 system [40]) to plants remains theoretical, as light-switchable kinase dimerization has not yet been demonstrated in planta [40,41]. In this conceptual design, the CPK32 kinase domain is fused with the PhyB photodomain, creating a light-activated system [40]. Red light (660 nm) would activate the system, while far-red light (730 nm) would inhibit it [40,41]. This would allow non-invasive, precise control of nitrogen assimilation in space and time. Note: This design is purely conceptual. No plant optogenetic kinase system has been published, and PhyB-PIF6 dimerization in plants has only been demonstrated for transcriptional control, not kinase activation [40,41].

##### Frequency Tuning Type

CPK Combinatorial Optimization: [HYPOTHESIS; AWAITS EXPERIMENTAL VALIDATION] Different CPKs may decode distinct calcium oscillation frequencies [47]. Using CRISPR, the signal integration characteristics of the nexus can be modified by adjusting the expression ratio of CPK10/30/32 [42,43,48,49], offering a method for optimizing response to varying nitrogen signals. Established fact: CPK10/30/32 show functional redundancy in NLP7 phosphorylation [9]. Hypothetical extension: Frequency-specific decoding by individual CPKs is a theoretical extrapolation from animal calcium signaling models [47]; no evidence supports frequency discrimination by plant CPKs.

#### 7.1.3. Layer 3: Output Layer Modification-Rational Optimization of Transcription Program

##### Evidence Status: [Partially Validated]

The following designs integrate proven nuclear retention mechanisms [6] with hypothetical spatiotemporal control elements. Synthetic promoter combinations have not been tested in planta.

The transcriptional activity of NLP7 is tightly regulated by its phosphorylation state and protein interactions [6,9,44], offering multiple targets for structure-guided engineering at the output layer (Layer 3, Figure 2).

##### Constitutive Activation

S205D phosphomimetic mutation: [Partially Validated] This mutation mimics the phosphorylation by CPK10/30/32 [9], enhancing NLP7 nuclear retention and transcriptional activation [6,9], leading to continuous activation of the primary nitrate response [6,9,24]. It is particularly suitable for maintaining growth under low nitrogen stress [24], but must be carefully integrated with carbon status monitoring to avoid an energy crisis [20]. Established fact: Ser205 phosphorylation is necessary and sufficient for nuclear retention [9]. Hypothetical extension: The S205D phosphomimic mutant has not been characterized in published studies; phenotypic predictions are extrapolated from CPK overexpression data [9] and phosphomimic studies in other transcription factors [50].

##### Space-Time Constraint

Synthetic promoter engineering: [Engineering Proposal; Untested] By combining four copies of the Nitrate Responsive Element (4 × NRE) [9] with tissue-specific promoters, spatially restricted nexus output can be achieved. For example, coupling with the endodermis-specific SCR [51] promoter or the root-cortex-specific NPF6.4 promoter ensures spatially restricted nexus output. Established fact: 4 × NRE synthetic promoters have been used in reporter assays [9]. Hypothetical extension: Their coupling with tissue-specific promoters for nexus engineering has not been experimentally validated; promoter choice is based on published expression patterns.

Growth stage-specific expression: [Engineering Proposal; Untested] Modifying the nexus to be active during critical growth stages can be accomplished using root-specific promoters that enhance nitrogen uptake during tillering (e.g., OsNAR2.1 promoter [52,53], which drives robust expression in root cortical cells during the vegetative and tillering stages) or shoot-tip meristematic tissue-specific promoters (e.g., KN1 promoter [54]). Note: Promoter choice is based on published expression patterns, but synthetic nexus constructs using these promoters have not been generated.

However, rational design must consider pleiotropic constraints: T101D may incur metabolic costs through excessive proton co-transport [8,12,36]; S205D risks anabolic imbalance if activated without carbon sufficiency (Section 4.4) [20,28]; and P492L may impair systemic nitrate translocation required for shoot cytokinin synthesis (Section 5.3) [4,5,27]. These constraints necessitate matched carbon-nitrogen engineering and tissue-specific promoters to mitigate off-target effects [20,42].

#### 7.1.4. Epistatic Constraints and Pleiotropic Risk Assessment

##### Evidence Status: [Predictive Modeling]

The following pleiotropic risk assessments are derived from published mutant phenotypes and metabolic pathway analysis but represent predictive extrapolations rather than experimentally validated epistatic interactions.

Structure-guided engineering of the nexus must account for layer-specific pleiotropic effects that may compromise agronomic performance.

##### Layer 1 Risks: T101D Metabolic Cost and T101A Nitrogen Deficiency

At Layer 1, T101D-mediated high-affinity locking enhances nitrogen foraging but may impose metabolic costs: excessive proton co-transport (2 H^+^ per NO_3_^−^) could deplete membrane potential and ATP reserves, particularly under low-carbon conditions where SnRK1 is activated [20,28]. [Prediction Based On: chl1-5 mutant phenotypes showing transport defects [8,12] + SnRK1-NLP7 interaction demonstrating carbon-nitrogen crosstalk [20,28]].

Conversely, T101A variants risk nitrogen deficiency signatures (chlorosis, stunted growth) in marginal soils with unpredictable nitrogen leaching, as demonstrated by chl1-5 mutant phenotypes [12,55]. [Validated Phenotype; Environmental Extrapolation].

##### P492L Systemic Effects: Root-to-Shoot Nitrate Translocation

P492L transport-signaling decoupling preserves calcium signaling but may disrupt root-to-shoot nitrate translocation required for systemic cytokinin synthesis via IPT3/ABCG14 (Section 5.3) [5,27,56], suggesting that partial-function alleles retaining 10–20% transport activity may be preferable to complete nulls. [Prediction Based On: P492L signaling retention [5] + IPT3/ABCG14 systemic pathway [27,56]].

##### Layer 3 Risks: S205D Constitutive Activation and Anabolic Crisis

At Layer 3, S205D constitutive activation carries the most severe pleiotropic risk: unregulated PNR activation independent of carbon status may trigger anabolic crisis, evidenced by SnRK1-mediated NLP7 degradation at Ser125/306 under energy deficit (Section 4.4) [20]. [PREDICTION BASED ON: SnRK1 phosphorylation sites + NLP7 degradation mechanism [20]].

##### Cross-Layer Epistasis: Synergistic Effects and Systems Modeling Requirements

Cross-layer epistasis further complicates prediction: Layer 1 (T101D)–Layer 3 (S205D) combinations may synergistically enhance nitrogen uptake but could exceed cellular assimilation capacity, necessitating matched enhancement of carbon fixation (e.g., PEPC overexpression [57]) or TOR pathway modulation [17,18]. [THEORETICAL PREDICTION; NO VALIDATED EXAMPLES] These considerations underscore the requirement for quantitative systems modeling, iterative greenhouse validation, and carbon-nitrogen status sensors as safety locks prior to field deployment [20,28].

### 7.2. Synthetic Reconstruction and Orthogonal System Verification

**Figure 3:**
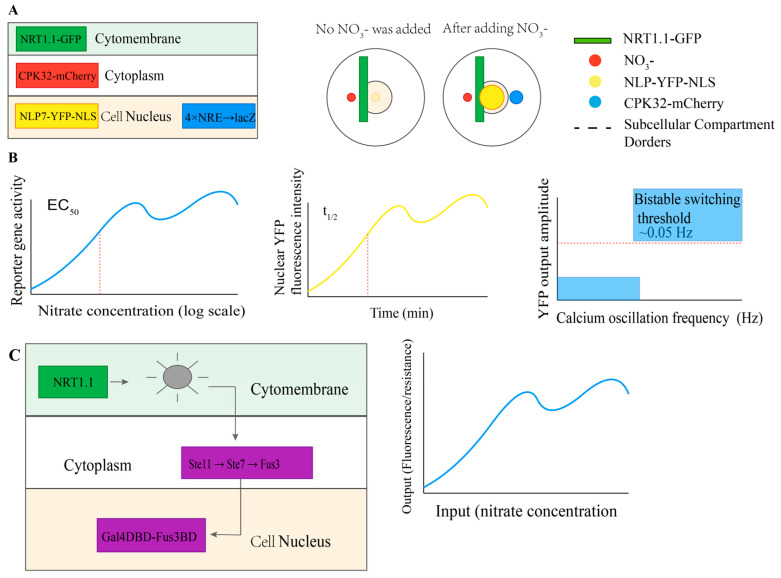
Synthetic reconstruction and orthogonal verification of the NRT1.1-NLP7 nexus in yeast. (**A**) Three-tier synthetic reconstruction system in Ogataea polymorpha Δynt1, comprising NRT1.1-sfGFP (sensing module), CPK32-mCherry (signaling module), and NLP7-YFP-NLS + 4 × NRE-lacZ (effector module). (Right): nitrate-induced nuclear import of NLP7 and reporter activation. (**B**) Verification metrics for the yeast chassis: nitrate dose–response curve (EC_50_), NLP7 nuclear import kinetics (t_1/2_), and calcium frequency-encoded output transfer function (bistable switching threshold ~0.05 Hz). (**C**) (Left) Orthogonal nexus coupling the NRT1.1 sensing domain to the yeast MAPK cascade (Ste11→Ste7→Fus3) with Gal4DBD-Fus3BD transcriptional output. (Right): dose–response output of the orthogonal system. Expanded plant cell validation using tobacco BY-2 cells (GCaMP6s, PP2A, TOR reporters) is described in Section 7.2.2.

To validate these engineering strategies and analyze information processing rules, synthetic reconstruction systems provide a powerful platform for dissecting the minimal functional unit of the NRT1.1-NLP7 nexus and screening for modulators in a high-throughput manner (Figure 3). This approach eliminates interference from endogenous plant pathways and enables precise quantification of signal transmission kinetics [38,58].

#### 7.2.1. Yeast Synthetic Reconstruction System

Chassis: *Ogataea polymorpha* (formerly *Hansenula polymorpha*, Δynt1 mutant, lacking endogenous nitrate transporter YNT1 and transcription factor YNA1) [59,60,61]. *O. polymorpha* is one of the few yeasts capable of utilizing nitrate, and its mutants have been used to verify plant NRT1.1 functionality [58].

Components:

Sensing module: *Arabidopsis* NRT1.1 (codon optimized, C-terminal fusion with sfGFP) [38,58];

Signaling module: CPK32 (cytoplasmic expression, fused with mCherry) [9];

Effector module: NLP7 (nuclear localization, fused with YFP-NLS) [6,9] + 4 × NRE-driven lacZ reporter gene [9];

Verification indicators: Nitrate dose–response curve (EC50), NLP7 nuclear input kinetics (t_1/2_), and frequency encoding properties (calcium oscillatory input-YFP output transfer function) [6,38,47,58].

#### 7.2.2. Tobacco BY-2 Cell Synthesis and Reconstruction System

Expanded components: GCaMP6s calcium indicator [13,62], PP2A regulatory subunit [39], and TOR activity reporting system [17,18].

Applications: Testing Layer 1–3 structure-guided engineering strategies in a plant cell environment [43] and high-throughput screening of modulators affecting nexus activity (chemical genetics) [43,48].

#### 7.2.3. Orthogonal Nexus

Design: Coupling the NRT1.1 sensing domain with the yeast MAPK cascade (Ste11 → Ste7 → Fus3) [63], further linked to a synthetic transcriptional output module (Gal4DBD-Fus3BD-driven reporter) [63], to create a plant-yeast hybrid signaling system.

Purpose: To analyze the information processing ability of the nexus in a simplified genetic background, eliminating interference from endogenous pathways; develop soil nitrate biosensors [64]; and conduct high-throughput screening of nexus-specific regulators [43,63].

### 7.3. Field Deployment Strategy and Risk Assessment

Translating laboratory-based structure-guided engineering into agricultural applications requires comprehensive environmental adaptability, biosafety, and economic viability.

#### 7.3.1. Environmental Adaptability Genotype Matching

**Figure 4:**
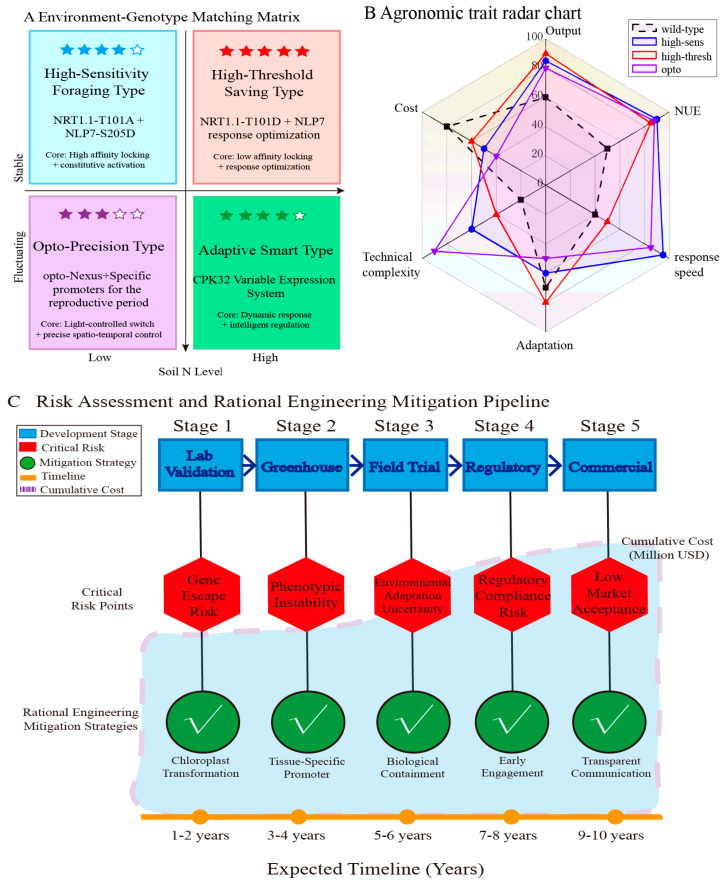
Field Deployment of Rationally Engineered Nexus Variants: Risk Assessment Framework. (**A**) Environment–genotype matching matrix. Quadrant plot of soil nitrogen availability (x-axis) vs. supply stability (y-axis). Four optimal engineering genotypes: high-sensitivity foraging type (**upper-left**, blue), high-threshold energy-saving type (**upper-right**, red), adaptive smart type (**lower-right**, green), and opto-precise type (**lower-left**, purple). Arrows indicate core rational design combinations; color intensity represents expected benefit magnitude. (**B**) Radar chart of agronomic traits. Six-dimensional comparison of wild type (dashed black) vs. engineered variants: high-sensitivity (solid blue), high-threshold (solid red), and optogenetic (solid purple). Dimensions: grain yield, NUE, response speed, adaptability, technical complexity, and cost. (**C**) Risk assessment and engineering mitigation workflow. Five-stage pipeline (laboratory validation → greenhouse testing → field trials → regulatory approva l → commercialization) with critical risk nodes (red hexagons) and mitigation strategies (green checkmarks). Bottom timeline shows expected duration and cumulative cost. Color-coded risk nodes correspond to evidence stratification: green checkmarks indicate Layer 1-validated mitigations.

To bridge the gap between computational design and agronomic application, we propose a genotype–environment–management (G × E × M) matching matrix that assigns structure-guided nexus variants to specific soil nitrogen scenarios. For nitrogen-deficient soils (<50 mg kg^−1^), a highly sensitive foraging genotype combining the NRT1.1-T101A high-affinity lock with root-specific constitutive activation of NLP7-S205D [9,12,38] is predicted to enhance nitrogen foraging while maintaining growth under low-input conditions. In high-fertility soils (200 mg kg^−1^), a high-threshold energy-saving genotype employing the NRT1.1-T101D low-affinity lock [12,38,55] reduces luxury nitrogen uptake and mitigates environmental nitrogen loss. Where nitrogen supply is temporally heterogeneous, an adaptive intelligent genotype utilizing a CPK32 variable-expression system [9,43,48] enables dynamic reprogramming of signal-computation kinetics, thereby matching nexus output to fluctuating nutrient availability, supported by precision-agriculture monitoring [65,66]. For controlled greenhouse environments, a light-controlled, precise genotype integrating an opto-Nexus system with growth-stage-specific promoters [40,41] offers on-demand activation of nitrogen assimilation via LED light control, although this Phase III concept remains contingent upon optogenetic breakthroughs in planta. Collectively, these environment-specific designs stratify engineering targets from validated Layer 1 alleles (Phase I) through model-based Layer 2 optimizations (Phase II) to theoretical Layer 3 implementations (Phase III), providing a phased roadmap for context-dependent nitrogen-use-efficiency improvement (Figure 4; Table 1).

#### 7.3.2. Biosecurity and Ecological Risks

To ensure the safe field deployment of rationally engineered nexus variants, we propose a tiered biosafety risk assessment framework that stratifies potential hazards by probability and severity, pairing each risk class with targeted engineering mitigation strategies (Table 1). For high-risk scenarios, transgene flow to wild relatives via pollen dispersal represents the most severe biosafety threat; this is addressed by chloroplast transformation, which enforces maternal inheritance and eliminates pollen-mediated gene escape. Moderate-risk off-target effects include disruption of mycorrhizal symbiosis or hormone balance, mitigated through root-specific promoters (e.g., RCc3) and chemically inducible systems (e.g., DEX-induced) that restrict transgene expression to target tissues. Similarly, moderate-risk ecological adaptation—specifically the potential for engineered genotypes to become invasive—is countered by conditional growth restriction requiring specific nutrient inputs and continuous field performance monitoring. Low-risk perturbations to the rhizosphere microbiome nitrogen cycle are managed through long-term orientation tests (>10 years) and synthetic colony recovery protocols, ensuring compositional shifts remain below the 10% threshold. Notably, Genetic Use Restriction Technology (GURT) is excluded from the mitigation portfolio due to international regulatory restrictions under the Cartagena Protocol on Biosafety [26]. Collectively, these mitigation measures provide a quantitative, evidence-stratified safety net that aligns with the phased deployment roadmap from laboratory validation to commercialization (Table 2).

#### 7.3.3. Economic Feasibility and Social Acceptance of Structure-Guided Engineering

Cost–Benefit Model (Taking Rice as an Example): The potential economic benefits of high NUE varieties in structure-guided engineering need to be verified through farm-scale trials. Referring to the commercialization experience of conventional genetically modified crops, the break-even point usually requires large-scale cultivation (50,000 hectares) and a stable premium market [67].

Social acceptance: Early participatory assessment and transparent communication are required. Distinguish the regulatory differences and public perception differences between “gene editing” (SDN-1/2, regulated as non-GMO in most countries) [26] and “genetically modified” (GMO) [26,67].

### 7.4. Conclusions and Future Perspectives

#### 7.4.1. Theoretical Challenges and Knowledge Gaps

Despite the integrative framework presented herein, several fundamental questions remain unresolved that constrain predictive engineering of the NRT1.1-NLP7 nexus.

##### Structural Elucidation of the Full Signaling Complex

While crystal structures of NRT1.1 [12] and AlphaFold2-Multimer predictions of NLP7 [9] provide atomic-resolution snapshots, the dynamic NRT1.1-CNGC15 membrane complex and downstream NLP7 nuclear complexes have not been structurally visualized in a unified framework. Cryo-electron microscopy of this membrane-associated, transient complex faces technical challenges: (i) the NRT1.1-CNGC15 interaction is ligand-dependent and transient, requiring stabilized conformations for particle averaging [8,70,71]; (ii) NLP7 is intrinsically disordered in its N-terminal regulatory region, complicating density interpretation [9]; and (iii) the complex spans membrane (NRT1.1-CNGC15) and soluble (NLP7) compartments, necessitating detergent-free membrane mimics (e.g., nanodiscs) that may alter interaction kinetics [70]. Alternative approaches, such as in-cell cryo-electron tomography or FRET-based structural mapping, may be required to capture physiologically relevant conformations [71].

##### Decoding Calcium Signal Specificity

The “calcium decoding” hypothesis posits that CPKs translate dynamic calcium signatures into specific phosphorylation outputs [13,47]. However, whether distinct calcium oscillation frequencies (e.g., 0.1 Hz vs. 1 Hz) differentially activate CPK10, CPK30, or CPK32 remains untested. Engineered calcium reporters with improved temporal resolution (e.g., GCaMP8 variants) and optogenetic calcium actuators (e.g., ChR2-CNGC chimeras) are needed to causally test frequency-dependent encoding [41,62]. Furthermore, the existence of CPK-independent phosphorylation pathways—potentially involving CIPK23 or MAPK cascades [9,14,15,16]—suggests that the calcium-CPK-NLP7 axis may be one of several parallel routes, complicating predictive modeling.

##### NLP7 Direct Sensing Versus Indirect Activation

The discovery that NLP7 binds nitrate directly [9] challenges the primacy of NRT1.1-mediated calcium signaling in nitrate perception. Critical unresolved issues include: (i) the affinity of NLP7 for nitrate (Kd) and whether this falls within physiologically relevant cytosolic concentrations (estimated 1–10 mM in root cells) [9]; (ii) whether nitrate binding to NLP7 occurs independently of or synergistically with CPK-mediated phosphorylation [9]; and (iii) the structural mechanism by which nitrate binding unmasks the nuclear localization signal [6,9]. Addressing these questions requires nitrate-binding assays with purified NLP7 (e.g., isothermal titration calorimetry) and phosphomimic/phosphodead mutants that disentangle allosteric and phosphorylation-dependent activation [9].

##### System-Level Feedback Integration

The nexus does not operate in isolation; it is embedded within carbon-nitrogen crosstalk (Section 4.4), hormone signaling (Section 5.2), and microbiome interactions (Section 7.3.2). Current models treat these as modular inputs, but emergent properties—such as how carbon starvation via SnRK1 overrides nitrate signaling [20] or how rhizosphere microbiome composition alters root nitrate sensing—remain poorly parameterized. Multi-omics time-series analysis (transcriptomics, metabolomics, phosphoproteomics) under fluctuating nitrogen-carbon conditions is needed to build dynamic, rather than static, regulatory models.

#### 7.4.2. Technical Bottlenecks and Methodological Innovations

Translating the structure-guided engineering framework from computational design to field-ready crops requires overcoming substantial technical barriers [25].

##### In-Planta Validation of Layer 2–3 Designs

While Layer 1 variants (T101A/D, P492L) can be directly introduced via CRISPR-Cas9 [72,73], Layer 2 strategies (CPK32 modulation [9], phosphatase inhibition [39]) and Layer 3 designs (synthetic promoters [42], optogenetic systems [40,41]) lack rapid validation platforms [25,43]. The yeast synthetic reconstruction system (Section 7.2.1) [58] and tobacco BY-2 cell assays (Section 7.2.2) [62,74] provide useful first-tier characterization, but these heterologous systems lack: (i) the complete complement of plant phosphatases that reset NLP7 activity [39]; (ii) the carbon-sensing machinery (SnRK1, TOR) that gates nexus output [17,18,20]; and (iii) the developmental context that determines cell-type-specific responses [51,54]. A “nexus-on-a-chip” microfluidic root system [75,76], combining controlled nitrate gradients with real-time biosensor readouts (e.g., NLP7-NLS-GFP nuclear import reporters) [6], could bridge this gap by enabling high-throughput phenotyping of engineered variants in a physiologically relevant context [43,48,77].

##### Optogenetic Implementation in Crops

The opto-CPK design (Section 7.1.2) remains theoretical because plant cells present unique obstacles: chloroplasts absorb red light (660 nm), potentially creating off-target activation [40]; phytochrome photoreversibility requires prolonged far-red illumination that may interfere with photomorphogenesis [40,41]; and tissue penetration of light in soil-grown roots is minimal [78]. Alternative strategies, such as chemogenetic approaches (e.g., rapamycin-inducible FKBP-FRB dimerization systems [79]) or thermogenetic actuators [80], may offer more practical spatiotemporal control for root engineering.

##### Multiplexed Genome Editing for Cross-Layer Epistasis

The pleiotropic risk assessment (Section 7.1.4) [20,28,57] predicts that combining Layer 1 and Layer 3 modifications (e.g., T101D + S205D) may cause anabolic crisis [20,57]. Testing these epistatic interactions requires simultaneous editing of multiple loci with predictable expression ratios. Current CRISPR-base-editing and prime-editing technologies enable precise single-nucleotide changes [81,82], but stacking multiple edits in cis (same chromosome) while maintaining independent regulatory control remains challenging. Synthetic biology approaches, such as artificial chromosome platforms or transgene landing pads [83,84], may be needed to assemble complex, multi-gene nexus cassettes with tissue-specific promoters [45,51].

##### Field-to-Laboratory Translation Gap

The risk assessment framework (Figure 4, Table 2) is based on ecological models and greenhouse data [68], but field performance depends on genotype × environment × management (G × E × M) interactions that are notoriously difficult to predict [85,86,87]. Nitrogen leaching, soil microbiome composition, and climate variability introduce noise that may mask engineered phenotypes [87,88]. Long-term, multi-site field trials (minimum 3 years, 5 locations) with isogenic lines differing only in nexus modifications are essential to distinguish signal from noise [68,89]. Such trials are resource-intensive and require public–private partnerships to share costs and germplasm [90].

#### 7.4.3. Integrative Outlook and Application Roadmap

We propose a phased roadmap for translating nexus engineering from basic discovery to agricultural deployment, aligned with the evidence stratification framework (Section 7.1).

##### Phase I (0–3 Years): Validation of Layer 1 Designs

Priority targets include T101D and T101A alleles in rice and Arabidopsis [4,5,12,29,38], characterized by nitrate uptake kinetics, root plasticity phenotyping [1,91], and preliminary yield trials under controlled nitrogen regimes. This phase leverages existing CRISPR infrastructure [73] and requires minimal methodological innovation. Success criteria: demonstration of >15% NUE improvement in greenhouse conditions without yield penalty under nitrogen sufficiency [29,30,52].

##### Phase II (3–7 Years): Layer 2 Optimization and Layer 3 Prototyping

Focus on CPK combinatorial optimization (CRISPR-mediated promoter replacement to tune CPK10/30/32 expression ratios) [9] and synthetic NRE promoter testing in model systems [10,23,60]. The yeast and BY-2 reconstruction systems (Section 7.2) [58,74] will be critical for high-throughput screening of signaling modulators. Success criteria: identification of CPK expression ratios that optimize response speed without causing response fatigue; demonstration of tissue-restricted nexus output using SCR or NPF6.4 promoters [42,45,46,51].

##### Phase III (7–12 Years): Multi-Layer Stacking and Field Deployment

Combine validated Layer 1 and Layer 2 modifications with carbon-status safety locks (e.g., SnRK1-sensitive NLP7 variants that deactivate under energy deficit) [20]. Conduct multi-site field trials with rigorous biosafety monitoring (Table 2) [68,89] and participatory assessment with farming communities [26,90]. Success criteria: stable NUE improvement (>20%) across diverse soil nitrogen conditions; no detectable off-target effects on mycorrhizal symbiosis or soil microbiome composition (<10% shift, Table 2) [68,89]; regulatory approval for gene-edited (SDN-1/2) varieties in target markets [26].

##### Convergent Opportunities: Fundamental Biology Tools Beyond Crop Improvement

Beyond crop improvement, the NRT1.1-NLP7 nexus framework offers tools for fundamental biology: (i) the synthetic reconstruction systems (Section 7.2) can be repurposed as soil nitrate biosensors for precision agriculture [64,92,93]; (ii) the dual-sensor architecture (Section 4.5) provides a template for understanding other transceptor-transcription factor nexuses; and (iii) the optogenetic and chemogenetic toolkits developed for nexus control may generalize to other plant signaling pathways [40,41,42,43,48]. These convergent applications underscore the value of investing in nexus-focused research, even as specific engineering strategies evolve.

Finally, we emphasize that the structure-guided engineering framework presented herein is stratified by evidence maturity. Layer 1 strategies (T101A/D, P492L) are immediately deployable for proof-of-concept studies given their grounding in validated mutant phenotypes [4,5,12,38]. Layer 2 proposals (CPK modulation, phosphatase targeting) require kinetic validation through in vitro phosphorylation assays and transgenic characterization before field application [9,39]. Layer 3 designs (synthetic promoters, optogenetic systems) remain theoretical and await technological breakthroughs in plant synthetic biology [42,43,48]. This tiered presentation is intentional: it distinguishes actionable engineering targets from exploratory hypotheses, guiding resource allocation in crop improvement programs and preventing premature deployment of unvalidated strategies.

## Figures and Tables

**Table 1:** Environment-specific structure-guided engineering designs for optimized nitrogen use efficiency.

Characteristics of Soil Nitrogen	Target Trait	Structure-Guided Engineering Genotype	Core Transformation Portfolio	Expected Benefits	Management Support	Development Phase
Low-nitrogen infertile soil (<50 mg/kg)	Enhance foraging	Highly sensitive foraging type	NRT1.1-T101D (High affinity locking) + NLP7-S205D [9,12,38] (constitutive activation, root specific)	Low nitrogen for increased production	Cooperate with diazotrophic bacteria inoculation	Phase I–II (Layer 1 validated; Layer 3 prototyping)
High nitrogen fertility (200 mg/kg)	Reduce the absorption of luxury	High-threshold energy-saving type	NRT1.1-T101A [12,38,55] (low affinity locking) + NLP7 response optimization	Nitrogen reduction potential	Reduce fertilization and optimize top dressing	Phase I (Layer 1 validated)
Fluctuating supply	Quick response	Adaptive intelligent type	CPK32 Variable Expression System [9,43,48]	NUE’s potential for improvement	Precision Agriculture monitoring [65,66]	Phase II (Layer 2 optimization)
Greenhouse Production	Precise regulation of the growth period	Light-controlled precise type	opto-Nexus system [40,41] growth-specific promoter	Activate on demand	LED light control system [40,41]	Phase III (Layer 3 theoretical; awaits optogenetic breakthroughs)

**Table 2:** Biosafety risk assessment and mitigation strategies for rationally engineered genotypes.

Risk Level	Type of Risk	Concrete Expression	Engineering Mitigation Strategies	Expected Outcome
High	Gene drift	Transgene flow to wild relatives	Threshold: >1% gene flow in 100 m [67]. Strategy: Chloroplast transformation (matrilineal inheritance reduces pollen dispersal) [65]	Maternal inheritance eliminates pollen-mediated gene flow; transgene containment achieved
Moderate	Off-target effect	Affecting mycorrhizal symbiosis or hormone balance	Threshold: 20–50% colonization reduction [68]. Strategy: Tissue-specific promoter (RCc3 [45]); chemically induced system (DEX-induced [69])	Root-restricted expression minimizes non-target organism impact; symbiosis deviation maintained <10%
Moderate	Ecological adaptation	Becoming invasive in the wild	Threshold: Competitive advantage index > 1.5 [68]. Strategy: Conditional growth restriction (requires specific nutrients); field performance monitoring	Nutrient-dependent growth prevents invasiveness; competitive advantage index restored to <1.0
Low	Microbiome perturbation	Microbial alterations in the rhizosphere nitrogen cycle	Threshold: <10% microbiome shift [68]. Strategy: Long-term orientation test (>10 years); synthetic colony recovery	Rhizosphere compositional shift maintained <10%; synthetic community recovery within 2 seasons

Note: Risk levels are stratified by quantitative thresholds: High (>1% gene flow probability within 100 m radius [67]); Moderate (20–50% phenotypic deviation in non-target organisms [68]); Low (<10% shift in core microbiome composition [68]). GURT (Genetic Use Restriction Technology) is excluded as a mitigation strategy due to international regulatory restrictions under the Cartagena Protocol on Biosafety [26]. Threshold values are derived from published ecological risk assessment frameworks [26,68]. Abbreviations: GURT, genetic use restriction technology; DEX, dexamethasone; RCc3, rice root-specific promoter.

## Data Availability

This is a review article, and no new data were generated or analyzed in this study. All data cited are from publicly available sources as referenced in the manuscript.
